# **Research on glass surface defect detection method based on shadowgraphy imaging and improved YOLOv11n**

**DOI:** 10.1038/s41598-026-56410-y

**Published:** 2026-06-04

**Authors:** Yulu Xue, Yang Li, Hongbin Chen, Jia Wang, Xuewei Wang, Lihui Wu

**Affiliations:** Beijing Institute of Graphic Communication, Beijing, 102600 China

**Keywords:** Glass defects, Deep learning, Defect detection, YOLOv11n, Engineering, Mathematics and computing

## Abstract

Addressing the challenges of observing and detecting defects on ordinary glass surfaces, this paper proposes a defect detection method combining shadowgraphy technology with the YOLO_FSA algorithm. By constructing a glass surface defect image dataset using a shadowgraphy system and adopting YOLOv11n as the base model, the C3k2_FWD module is introduced to achieve efficient lightweight spatial-channel feature extraction and detection of elongated defects.A GSCSA attention mechanism is constructed to enhance perception of faint defect features. ADown convolution replaces traditional downsampling, achieving model lightweighting while maintaining detection accuracy. Experimental results show that the YOLO_FSA algorithm achieves precision, recall, and mAP50 of 74.8%, 80.7%, and 85.5%, respectively. Compared to YOLOv11n, these metrics improved by 8.5%, 6.4%, and 12.6%, respectively. Concurrently, the model parameters were reduced by 31.4% to 1.77 million, and computational complexity decreased by 25.4% to 4.7 GFLOPs, achieving an effective balance between detection performance and computational efficiency.

**Supplementary Information:**

The online version contains supplementary material available at 10.1038/s41598-026-56410-y.

## Introduction

 As a critical foundational material in displays, optical components, and new energy sectors, the surface and internal quality of glass directly determines the performance and reliability of end products. With the continuous elevation of quality requirements in high-end manufacturing, achieving efficient and precise detection of glass defects has become a core element of quality control^1^. However, the transparent, highly reflective, and low-contrast nature of glass makes surface defects such as scratches, contamination, and chipping extremely challenging to detect under conventional imaging conditions. These defects often exhibit minimal contrast with the background and blurred edges^2^.

Traditional glass defect detection primarily relies on image processing techniques for feature extraction and classification^3^, such as binarization, edge detection, and morphological operations. While these methods enhance detection efficiency to some extent, their reliance on manually designed features results in poor generalization capabilities when confronting diverse defect morphologies, varying scales, and complex optical interference in industrial settings. This makes it difficult to meet the demands for stable and robust detection.

The origin and development of shadowgraphy technology have undergone a long journey.Its earliest roots date back to the 17th century, when British scientist Robert Hooke conducted experiments using a convex lens and two candles. One candle served as the light source, while the lens allowed observation of refractive disturbances caused by heated air around the flame of the other candle—considered the technology’s prototype^4^.In the 19th century, German physicist Toepler made significant innovations. He introduced adjustable slit masks, dedicated light sources, and observation telescopes, constructing a structurally complete shadowgraph apparatus that became the foundation for modern shadowgraph systems. Subsequently, fellow German scholar Mach applied shadowgraph technology to visualize shock waves, greatly advancing the development of gas dynamics.Entering the 20th century, researchers like Schardin established a systematic theoretical framework for shadowgraph technology. Since then, it has been widely applied in critical fields such as ballistics, rocket technology, convection studies, combustion science, and wind tunnel experiments, becoming a key method for flow visualization and optical diagnostics. The core capability of shadowgraph systems lies in their ability to make the “invisible” visible.Impurities on the glass surface cause minute variations in local refractive index. When parallel light passes through the glass, these refractive index gradients induce extremely subtle deflections in the light rays. The shadowgraph system employs a blade placed at the beam’s focal point to perform “spatial filtering.” This selectively blocks the undeflected background light while allowing light deflected by defects to pass through, ultimately forming a high-contrast image on the imaging plane. This process is akin to “photographing” light, directly revealing microscopic perturbations in the glass’s internal uniformity. It represents a non-contact, highly sensitive optical inspection method. Compared to other techniques, the shadowgraph system’s key advantage lies in its suitability for large-area, rapid inspection in industrial settings. While traditional microscopic observation methods (such as phase contrast microscopy) offer high precision, they suffer from limited field of view and slow speed.In contrast, the shadowgraph system can image an entire glass sheet at once, enabling near-real-time observation. Its relatively simple structure and high design flexibility make it particularly suitable for in-line quality control of materials like optical glass and filters. It efficiently detects internal non-uniform defects invisible to the human eye or even conventional machine vision systems, playing an irreplaceable role in ensuring the optical performance of transparent materials.

In recent years, deep learning-based object detection algorithms have made significant strides in industrial vision. TANG et al.^5^ created an optical lens defect dataset and achieved excellent recognition results by detecting surface and internal defects in optical lenses using an improved YOLOV5 model. Using Faster R-CNN^6^.

and the YOLO series^7–10^, have gradually replaced traditional methods as mainstream approaches due to their robust feature learning capabilities. Among these, the YOLO series algorithms are widely used in various defect detection tasks because of their good balance between speed and accuracy.However, existing general-purpose detection models still face significant limitations when applied to special materials like glass. On one hand, their insufficient perception of low-contrast, minute, and elongated defects leads to high false-negative rates^11,12^.On the other hand, the models’ large parameter counts and high computational complexity make deployment on resource-constrained industrial field devices challenging.Furthermore, the lack of specialized imaging solutions and data augmentation mechanisms tailored to glass optical properties also limits the model’s robustness in practical applications.

To address these challenges, this paper proposes a glass defect detection method based on a shadowgraph optical imaging system and designs an improved YOLOv11 model—YOLO_FSA—to enhance detection performance under low-contrast and blurred boundary conditions.First, a high-sensitivity reflective conical fringe optical system is constructed to convert glass surface defects into high-contrast visual images, thereby building a high-quality defect dataset. Second, based on YOLOv11n, the C3k2_FWD module is designed with partial convolution, efficient channel attention, depthwise separable convolution, and hierarchical residual fusion, enhancing the extraction capability of slender defects and weak features. Then, the GSCSA attention mechanism is introduced to improve the model’s perception of defect regions in complex backgrounds. Finally, the ADown lightweight downsampling module is adopted to significantly reduce model complexity while maintaining detection accuracy, meeting industrial edge deployment requirements.

Experimental results demonstrate that YOLO_FSA achieves an mAP50 of 85.5% on a self-built glass defect dataset, with detection accuracy and recall reaching 74.8% and 80.7%, respectively. Compared to the original YOLOv11n model, mAP50 improves by 12.6% points, with a 31.4% reduction in parameters and a 25.4% decrease in computational complexity, achieving a favorable balance between detection performance and computational efficiency. This method provides the glass manufacturing industry with an efficient, lightweight, and deployable intelligent defect detection solution, demonstrating promising engineering application prospects.

## Dataset acquisition and establishment

### Schlieren optical observation system

**Fig. 1:**
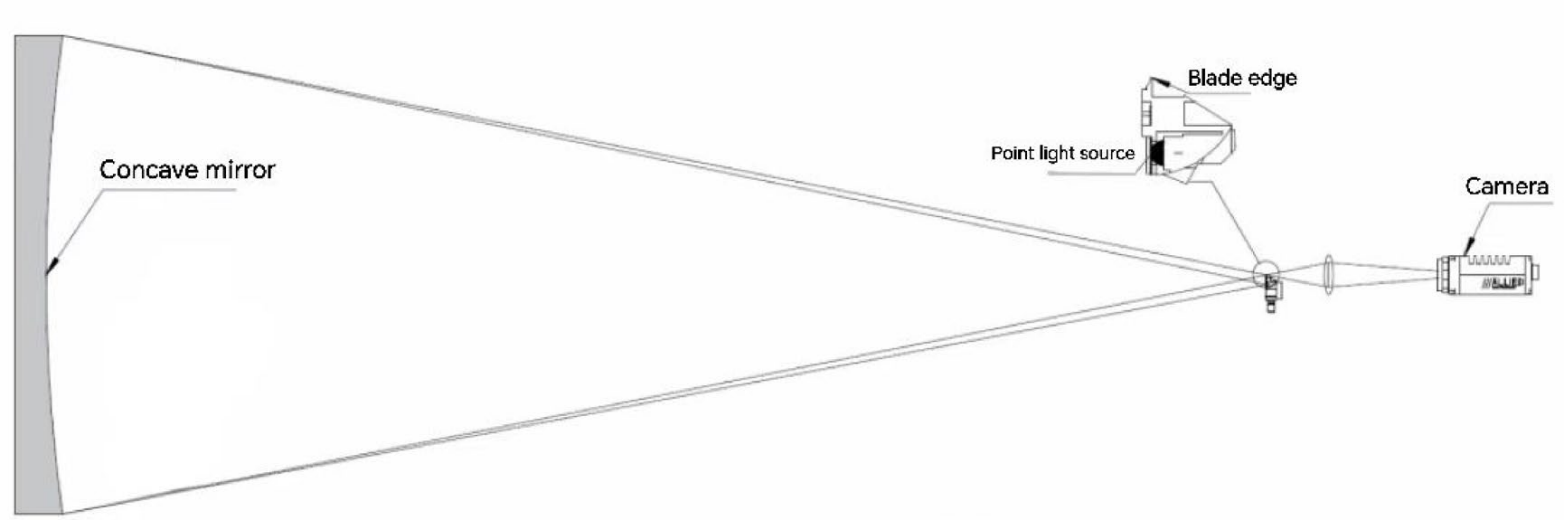
Optical path of conical light pattern system.

The Schlieren method, also known as the shadowgraphic technique, is a classical optical visualization technology. Its fundamental principle exploits the fact that the refractive index gradient in a measured flow field is proportional to the flow density. This converts density gradient variations within the flow field into relative intensity changes on the recording plane, rendering regions with intense density fluctuations—such as shock waves and compression waves in compressible flows—observable and distinguishable as images for subsequent recording.Combining high-speed cameras with high temporal resolution capabilities with the Schlieren method yields high-speed Schlieren imaging. Schlieren apparatus is simple in design and easy to operate, finding extensive applications in fields such as jet engine exhaust, flame combustion, and electric arc discharge^13–15^.Pattern projection systems are categorized into two main types based on the shape of the light path through the measured flow field: parallel-beam systems and conical-beam systems^16^. However, both share the same imaging principle. This experiment employs a conical light shadowgraph system, which features a simpler structure and higher sensitivity than parallel light shadowgraph systems. The optical path of the reflective conical light shadowgraph system is shown in Fig. 1.

### Defect image acquisition

**Fig. 2:**
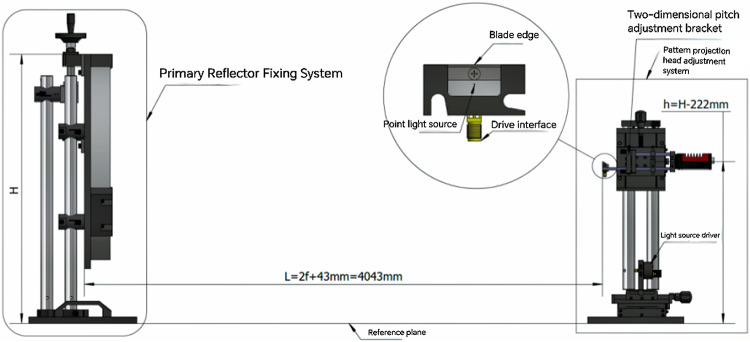
Mechanical diagram of conical optical pattern shadow.

The glass defect image acquisition in this study relies on a high-precision reflective conical Schlieren system. The entire apparatus is constructed using the Schlieren-V1.1 model designed by Ruiguang Kaiji (Zhenjiang) Optoelectronic Technology Co., Ltd. Its core principle involves passing the light beam twice through the glass sample under test, transforming originally transparent internal or surface flaws into highly visible, high-contrast Schlieren images. The system employs a 406 mm-aperture, 2000 mm-focal-length parabolic aluminum-coated mirror as the primary mirror. Combined with a 525 nm green LED point light source and a 50 μm slit, it generates a conical parallel light path within a fixed 4043 mm spacing.Positioned at the secondary focal point, the knife edge precisely blocks half the reflected light via a one-dimensional lift stage. This lowers the refractive index gradient response threshold to 1 × 10^-6^°, amplifying any minute density or thickness variation into sharply defined light-dark striations. The camera used is Daheng MARS-503-23GM-P, featuring 5.0 megapixels and 12-bit RAW output. Exposure time is locked at 1048 microseconds to prevent compression loss. The sample is vertically suspended 100 mm in front of the main mirror and fixed on an optical platform to prevent vibration. Experiments are conducted on an air-bearing optical platform, with the mirror wiped unidirectionally with anhydrous ethanol before each round of experiments. The experimental setup is shown in Fig. 2.

Illumination intensity and illumination mode have a significant influence on defect detection rate, especially in the surface inspection of transparent materials. Studies have shown that non-uniform illumination reduces the detection stability of deep learning models. The shadow imaging system used in this experiment employs a fixed 525 nm green LED point source as the sole illumination source. This wavelength exhibits favorable transmission properties in glass materials, and monochromatic light helps avoid interference from chromatic aberration on imaging. The point source is shaped by a 50 μm slit to form a highly collimated conical beam, whose output power is calibrated to remain stable within ± 2% of the set value, ensuring consistent illumination intensity. The illumination direction forms a fixed angle with the normal to the sample surface. Combined with the reflective optical path design of the concave mirror, this arrangement effectively prevents specular reflected light from directly entering the camera, thereby suppressing background noise. Ambient light is completely shielded during the experiment, and the imaging environment is determined solely by the system illumination, ensuring that the contrast of the captured images originates only from refractive index gradient changes caused by glass defects. The above illumination parameters were optimized through preliminary experiments, which can guarantee the clarity of defect edges while avoiding overexposure or underexposure that affects feature extraction, providing high-quality input data for the stable training of subsequent deep learning models.

Under these conditions, a total of 1,144 holograms at 2448 × 2048 resolution were captured (Fig. 2), covering three defect categories: scratches, chipped edges, and contamination, with over 300 samples per category.Each target in the images was annotated and classified using the LabelImg annotation tool. Data augmentation was then applied with Mixup α = 0.3 and a Mosaic probability of 0.5. This process yielded a high-fidelity, traceable glass defect dataset, establishing a reliable foundation for subsequent training and validation of the YOLO_FSA model.

The effectiveness of glass surface defect detection largely depends on an understanding of their physical origins. The three types of defects focused on in this study—scratches, contaminants, and edge defects—are the most representative defect types encountered during glass manufacturing and processing, and they exert the most significant impact on product quality.

Scratches mainly arise from mechanical contact between glass and hard objects, appearing as slender, continuous groove-like damages with depths ranging from micrometers to tens of micrometers. Optically, they cause abnormal scattering and refraction of incident light, with extremely low contrast against the background. In the shadow imaging system, the local refractive index gradient variations induced by scratches are amplified into distinct bright-dark fringes, enabling effective detection. Contaminants include foreign substances such as dust, oil stains, and fingerprints adhering to the glass surface, mostly introduced from the production environment. The refractive index difference between these contaminants and the glass substrate generates obvious optical contrast, which manifests as grayscale variations converted from phase modulation effects in the shadow imaging system. The randomness in the shape, size, and distribution of contaminants places high demands on the robustness of detection algorithms.Edge defects refer to local chipping or corner breakage of glass caused by mechanical impact or stress concentration. Their physical nature is brittle fracture, with irregular wedge-shaped or shell-like fracture morphologies. In optical inspection, edge defects are located at the glass boundary, and the scattering effect of the fracture surface appears as high-contrast anomalies in the shadow imaging system.

The above three defect categories are highly typical in the glass manufacturing industry, accounting for more than 80% of all glass surface quality issues, and serve as key criteria for product grading. In this study, these defects are imaged with high sensitivity via a shadow imaging system, and targeted feature extraction modules are designed (e.g., dynamic snake convolution for slender scratch detection and multi-scale feature fusion for identifying contaminants of varying sizes), fully accounting for the physical optical properties and geometric morphological characteristics of each defect type.

The classification of glass defects and the corresponding detection requirements involved in this study are mainly based on relevant national standards, including Flat Glass (GB 11614−2022) and Safety Glass for Building–Part 2: Tempered Glass (GB 15763.2–2005)^17,18^. Within the framework of these standards, the defect classification in this study follows the definitions specified in GB 11,614 − 2022, categorizing glass surface defects into three types: scratches, contaminants, and edge defects.Regarding the determination of defect dimensions, the standards require the detection and grading of defects whose length or area exceeds specified thresholds. To avoid ambiguity and overlap between different defect types, all samples were judged and screened by professional glass production technicians. Defects defined as detection targets in this paper are those with a contaminant area ≥ 0.5 mm^2^, an edge chipping width ≥ 0.5 mm, and a depth ≥ 1 mm, which were organized into a standardized dataset.

The shadow imaging system adopted in this study provides micrometer-level spatial resolution, enabling accurate measurement of defect geometric sizes and meeting the precision requirements for defect dimension inspection specified in the standards. The detection results output by the YOLO_FSA algorithm include defect category, location, and bounding box information. Combined with the conversion relationship between pixel size and actual physical size, quantitative evaluation of defect dimensions can be further realized, thereby aligning with the quality grading requirements stipulated in the standards.

## Algorithm improvements

**Fig. 3:**
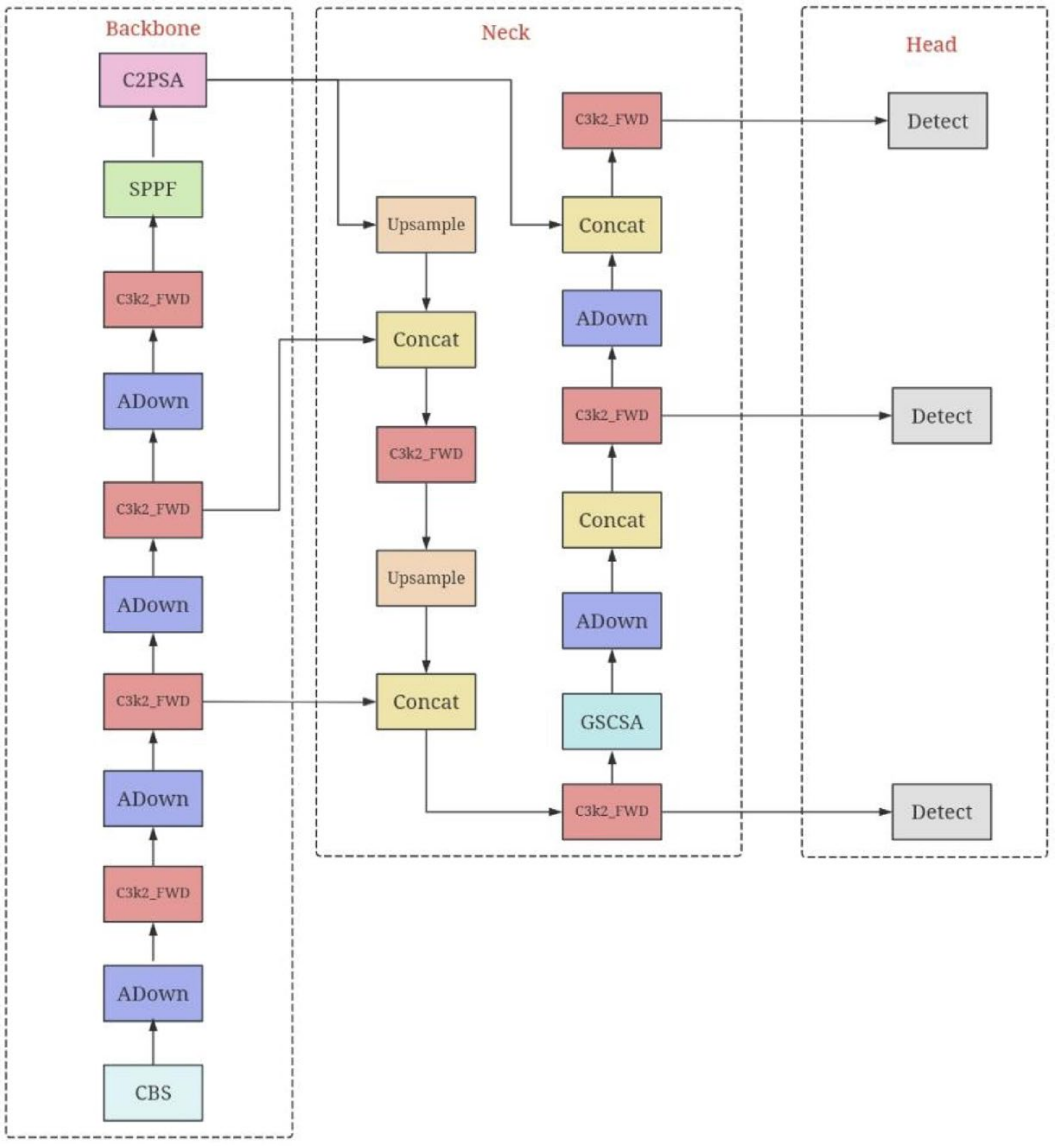
YOLO_FSA network architecture diagram.

Aiming at the characteristics of glass defect images captured by the schlieren optical observation system and the limitations of YOLOv11n in this scenario, and drawing on the optimization and application experience of YOLOv11 across multiple scenarios as well as the development characteristics of its overall framework, this paper proposes an improved YOLO_FSA (YOLO with FasterBlock, Spatial Channel Self-Attention and ADown) algorithm^19–21^. This algorithm achieves overall performance enhancement through the synergistic design of three key modules: First, the C3k2_FWD module integrates partial convolution, efficient channel attention (ECA), depthwise separable convolution, and hierarchical residual fusion to perform lightweight spatial-channel feature extraction and detail enhancement for glass defects.Second, the GSCSA attention mechanism is constructed to improve the perception of weak defect features through multi-scale feature fusion and enhanced spatial channel attention.Finally, ADown convolution replaces traditional downsampling methods, achieving model lightweighting while maintaining detection accuracy. The improved YOLO_FSA network architecture is shown in Fig. 3.

### C3k2_FWD module

**Fig. 4:**
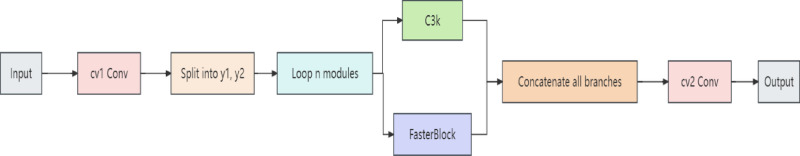
C3k2_FWD network architecture diagram.

For glass surface defect detection, this paper proposes an innovative C3k2_FWD module, whose overall architecture is illustrated in Fig. 4. Building upon the traditional C3k2 architecture^22^, the module incorporates multidimensional lightweight enhancements to establish a hierarchical, high-efficiency feature processing pipeline that balances detection accuracy with inference efficiency, thereby meeting real-time detection requirements in industrial applications.

First, replace the bottleneck structure (Bottleneck) with the FasterBlock to form C3k2_FasterBlock, which serves as the foundational unit of the C3k2_FWD module. The FasterBlock employs Partial_conv3 for efficient spatial feature mixing^23^, dividing the feature channels into a convolution-enhanced branch and an identity-retention branch, thereby performing spatial modeling only on selected channels while preserving global dimensional information and significantly reducing computational redundancy. The correspondence between the core channel splitting and fusion processes is illustrated in Formula (1):

1 $$\:\begin{array}{c}X=[{\mathrm{X}}_{1},{\mathrm{X}}_{2}],\hspace{1em}{\mathrm{X}}_{1}=Partial\_conv3\left({\mathrm{X}}_{1}\right),\hspace{1em}X=Concat({\mathrm{X}}_{1},{\mathrm{X}}_{2})\end{array}$$

In the formula, $\:{X}_{1}$ represents the feature channel involved in spatial mixing, while $\:{\mathrm{X}}_{2}$ denotes the feature channel retained directly. Partial_conv3 employs a group-depth convolution combined with a 1 × 1 convolution cascade architecture, enabling lightweight local feature extraction while maintaining consistent receptive fields and feature resolution.

To enhance the representation ability of defect-related channels, this paper deeply embeds the Efficient Channel Attention (ECA) mechanism into the MLP branch of FasterBlock, constructing a compact nonlinear structure of dimension augmentation, channel recalibration and dimension reduction.Unlike the dynamic kernel size strategy adopted by the conventional ECA, this paper conducts task-oriented optimization on the adaptive hyperparameters of kernel size according to the prior distribution of channel responses of glass defect data. This makes the attention receptive range steadily match the distribution of defect features and suppress training fluctuations induced by dynamic kernel size adjustment. The calculation process of channel attention is illustrated in Formula (2):

2 $$\begin{array}{l}\mathrm{Y}=\mathrm{A}\mathrm{v}\mathrm{g}\mathrm{P}\mathrm{o}\mathrm{o}\mathrm{l}(\mathrm{X}), \quad \mathrm{Y}=\mathrm{C}\mathrm{o}\mathrm{n}\mathrm{v}\mathrm{1}\mathrm{d}(\mathrm{Y})\quad \widehat{\mathrm{X}}= \mathrm{X} \odot \sigma \mathrm{(}\mathrm{Y}\mathrm{)}\end{array}$$

In the formula, $\:\left(\sigma\:\right)$ denotes the Sigmoid activation function. This architecture eliminates dimensionality reduction loss, with negligible additional parameters, enabling adaptive enhancement of channel responses corresponding to targets such as scratches and bubbles while suppressing substrate texture and lighting interference.

To further enhance lightweight performance, the C3k2_FWD architecture fully employs deeply separable convolutions and incorporates a unified autopad mechanism, ensuring strict scale alignment of feature maps before and after convolution for seamless integration with Partial_conv3. The deeply separable convolutions decouple standard convolution operations into channel-wise spatial filtering and point-wise channel fusion, significantly reducing computational complexity.Its calculation formula is shown in Formula (3):

3 $$\:\begin{array}{c}{{X}}_{{d}}= {D}{W}{C}{o}{n}{v}{(}{X}{,}{k}{,}{s}{,}{p}{)}, \quad \widehat{{X}}={P}{o}{i}{n}{t}{C}{o}{n}{v}{(}{{X}}_{{d}}{)}\end{array}$$

In the formula, DWConv denotes deep convolution, while PointConv represents a 1 × 1 point-by-point convolution. The padding parameter p is dynamically calculated by autopad based on the convolution kernel, stride, and expansion rate. Within the module, the deep separable convolution handles channel projection and global feature transformation, whereas Partial_conv3 manages local spatial mixing. Together, these components form a complementary and efficient lightweight feature extraction architecture.

Building on this foundation, C3k2_FWD introduces hierarchical residual connections and the Layer Scale mechanism to stabilize gradient propagation in deep networks, enhancing training stability and model generalization. The architecture employs a forward propagation strategy of splitting–progressive refinement–fusion: cv1 performs channel dimension splitting, the FasterBlock sequence achieves multi-level feature refinement, and cv2 ultimately performs multi-branched feature concatenation and dimension mapping. Residual fusion and overall forward propagation are described by Formula (4).

4 $$\:\begin{array}{c}\widehat{{X}}={X}{+}{D}{r}{o}{p}{P}{a}{t}{h}{(}\lambda{*}{M}{L}{P}{(}{X}{)})={c}{v}{2}\left({Concat}\left({{FasterBlock}}_{{i}}\left({C}{h}{unk}{(}{cv}{1(}{X}{))}\right)\right)\right)\end{array}$$

In the formula, $\:\lambda$ is the learnable scale coefficient, DropPath represents random path dropout regularization, and the MLP branch embeddings with ECA achieve channel augmentation and nonlinear transformations.

C3k2_FWD integrates partial convolution, efficient channel attention, depthwise separable convolution and hierarchical residual fusion modules. It is not a simple superposition of technologies, but a collaborative and hierarchical overall design tailored to the spatial morphology, frequency domain characteristics and industrial deployment constraints of glass defects. Partial_conv3 achieves efficient fusion of sparse textures with minimal computational cost. The ECA module realizes defect feature channel enhancement and background noise suppression. Depthwise separable convolution balances the capability of multi-scale feature extraction and actual inference efficiency. The hierarchical residual structure combined with the multi-branch fusion mechanism ensures the stable propagation and sufficient interaction of deep features.

The overall module follows the progressive information processing logic of dimension splitting, spatial mixing, channel enhancement, scale calibration and multi-branch fusion. The preceding components provide feature foundations with strong decoupling capability and high discriminability for subsequent modules. The subsequent modules further refine, strengthen and calibrate the pre-extracted features. All functional units cooperate synergistically, ultimately realizing efficient, accurate and robust feature extraction and representation for glass surface defects.

### GSCSA network architecture

**Fig. 5:**
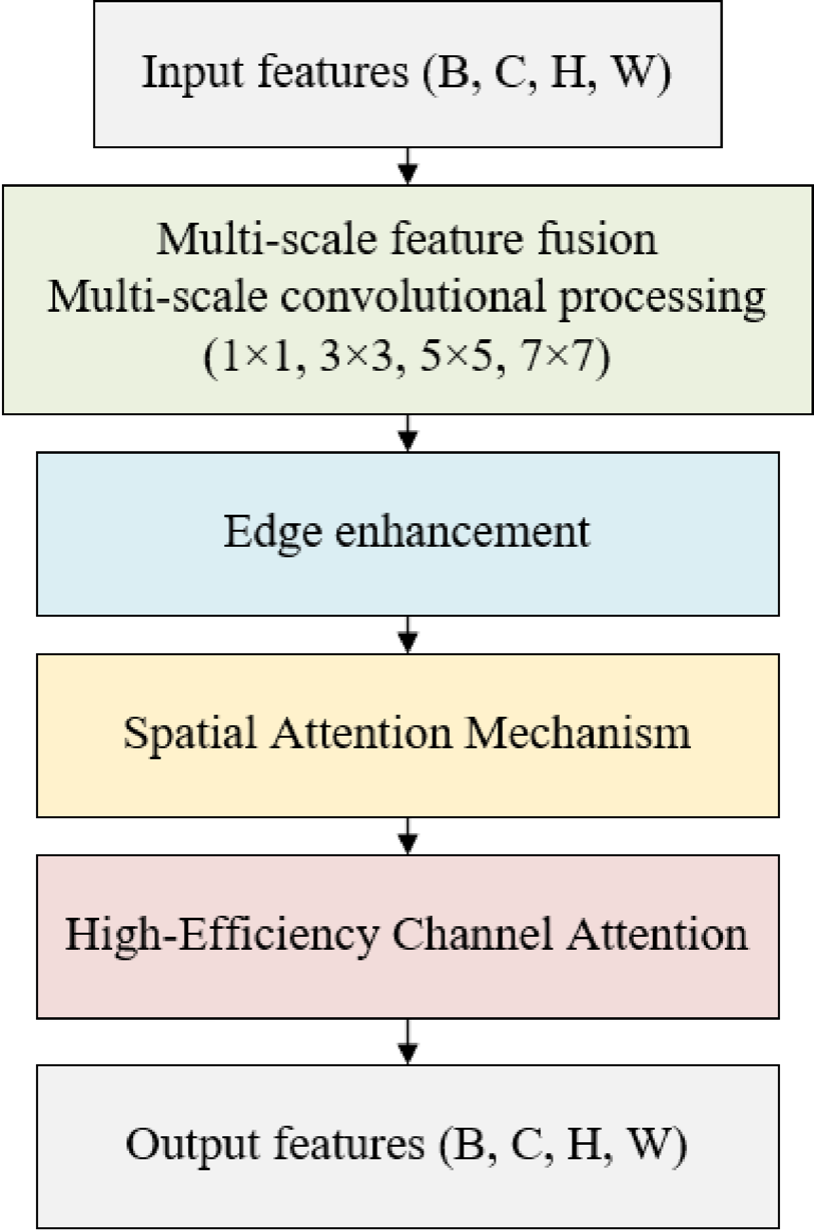
GSCSA network architecture diagram.

Optimized for detecting glass defects like scratches and stains, the GSCSA (Glass-oriented Spatial Channel Self-Attention) module is built upon the SCSA module. This paper integrates the GSCSA module into the small object detection layer of the YOLOv11 network. The GSCSA module adopts a lightweight design philosophy, comprising the following key components: Multi-Scale Feature Fusion, Efficient Channel Attention, Spatial Attention, and Edge Enhancement. The overall architecture achieves multidimensional enhancement of glass defect image features acquired by the optical system through residual connections and adaptive weight fusion. Its network structure is illustrated in Fig. 5.

Specifically, the MultiScaleFeatureFusion module employs four convolutional kernels of different scales (1 × 1, 3 × 3, 5 × 5, 7 × 7) to process input feature maps in parallel^23^:The 3 × 3 convolution handles local features, capturing small-scale scratches; the 5 × 5 convolution captures medium-range spatial information, identifying medium-sized defects such as stones; the 7 × 7 convolution focuses on global contextual information, addressing large-area optical inhomogeneities. This design is particularly suited to the imaging characteristics of shadow-imaging optical systems, effectively addressing variations in defect appearance caused by different refractive index changes.After deep separation via convolutional processing, features across scales undergo channel concatenation. Feature fusion is achieved through a 1 × 1 convolution and batch normalization layer, as illustrated by the multiscale feature fusion process in Eq. 5.

5 $${\mathrm{F}}_{{{\mathrm{multi}}}} = {\mathrm{Conv}}_{{1 \times 1}} ([{\mathrm{F}}_{1} ,{\mathrm{F}}_{3} ,{\mathrm{F}}_{5} ,{\mathrm{F}}_{7} ])$$

Here, ${\mathrm{F}}_{\mathrm{k}}$ denotes the convolutional output feature with kernel size k × k, and [] represents the channel dimension concatenation operation.

Additionally, the efficient channel attention mechanism combines the advantages of average and max pooling, computing channel weights^24^ through a shared multi-layer perceptron (MLP) network. This module employs a high channel reduction ratio (reduction = 8) to minimize parameters while ensuring a minimum channel count of 8, thereby preserving the ability to represent weak optical signals in ghost images.For high-contrast edge information generated by the holographic optical system, the MLP network comprises two 1 × 1 convolutional layers and a ReLU activation function. This architecture adaptively enhances defect-related channel features, ultimately generating channel attention weights via a Sigmoid function.

Notably, the spatial attention mechanism represents the core innovation of the GSCSA module for shadowgraph optical defect detection, employing a separated height and width dimensional processing strategy. Since shadowgraph systems are sensitive to refractive index gradients within glass, defects often exhibit specific spatial distribution patterns. Input feature maps are first subjected to average pooling along the height and width dimensions, yielding two one-dimensional feature vectors.Features from each dimension are grouped into four sets, each processed with one-dimensional convolutions using different kernel sizes (default: 3, 5, 7, 9). This grouped approach maintains computational efficiency while capturing spatial dependencies across different scales in the ghosting image. Processed features are normalized via GroupNorm, then spatial attention weights are generated using Sigmoid or Softmax activation functions. The height and width attention weights are multiplied via broadcasting to form a two-dimensional spatial attention map^25^. The spatial attention calculation is shown in Eq. 6:

6 $$\:\begin{array}{c}{{A}}_{{spatial}}=\sigma {(}{{A}}_{{h}} \otimes {{A}}_{{w}}{)}\odot{{F}}_{{input}}\end{array}$$

Where $\:{\mathrm{A}}_{\mathrm{h}}$ and $\:{\mathrm{A}}_{\mathrm{w}}$ represent the attention weights for the height and width dimensions, respectively. $\:\otimes$ denotes broadcast multiplication, *⊙* denotes element-wise multiplication^26^, and *σ* denotes the activation function.

Finally, to better detect linear defects like glass scratches displayed in the holographic optical system, the GSCSA module incorporates a dedicated edge enhancement module. Since holography itself is based on the principle of edge detection through light deflection, this module employs a 3 × 3 depth-separated convolution with batch normalization and ReLU activation to further accentuate existing edge information in the hologram image, enhancing response to faint optical signals.Edge features are connected to the main features via residual connections with smaller weights (0.05), preventing noise issues caused by excessive enhancement.

### ADown convolution

In the evolution of YOLO algorithms, the ADown module emerged as a lightweight design in YOLOv9^27,28^.It was further integrated into the YOLOv11 model in this study, replacing all convolutions except the first layer. This module focuses on optimizing the downsampling process, achieving efficient dimensionality reduction and feature extraction while preserving critical information.By adjusting the convolutional structure and stride settings, ADown enhances the overall simplicity and expressive power of the architecture while controlling model complexity.

**Fig. 6:**
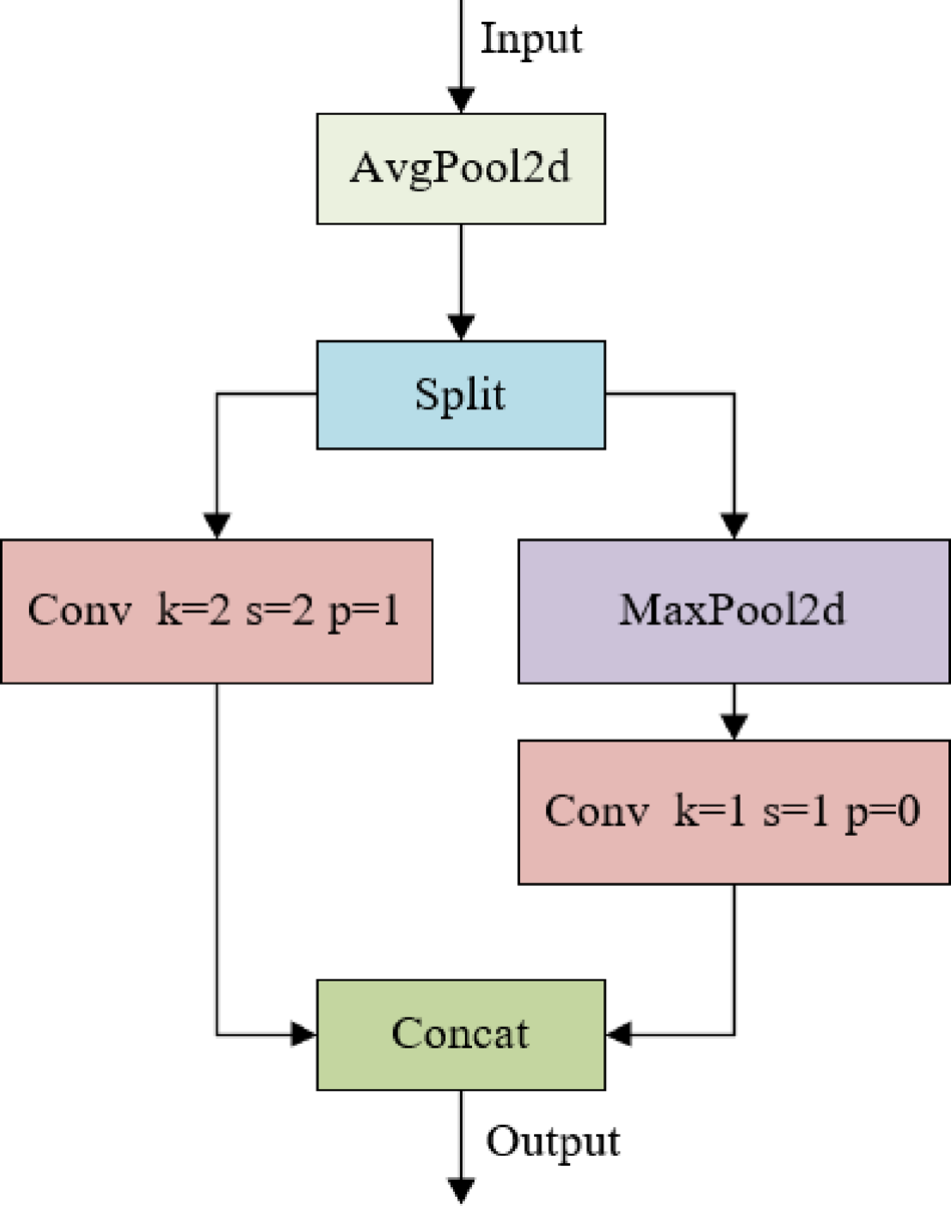
ADown network architecture diagram.

Structurally, the ADown module integrates multiple downsampling strategies including average pooling, max pooling, and convolution, maintaining stable channel counts while reducing spatial resolution. Its learnable parameters enable adaptive adjustment of downsampling strategies according to task requirements, demonstrating strong generalization capabilities. Compared to conventional downsampling methods, ADown significantly improves computational efficiency and reduces resource consumption while preserving detection accuracy. Figure 6 illustrates the specific structural design of this module.

## Experiments and analysis

### Experimental environment and parameter configuration

A comprehensive experimental environment was established with detailed parameter configurations, as shown in Table 1. The experimental platform utilized the Ubuntu operating system, PyTorch 2.0.0 as the deep learning framework, and Python 3.8.10 for code implementation. Hardware specifications included an RTX 4090 GPU for acceleration and a Xeon Platinum 8352 V processor, capable of meeting computational demands for large-scale network training.

**Table 1:** Experimental environment and parameters.

Environment Item	Environment Configuration	Parameter	Parameter Configuration
Operating System	Ubuntu	Epoch	200
Deep Learning Framework	PyTorch 2.0.0	Batch Size	64
Programming Language	Python 3.8.10	Image Size	640 × 640
GPU	RTX 4090	lr0	0.01
CPU	Xeon Platinum 8352 V	Optimizer	SGD

### Dataset and evaluation metrics

The glass defect dataset used in this study was collected via a shadowgraphy optical observation system and annotated using the Labelimg tool, comprising a total of 1,144 high-quality glass defect images. The dataset was divided into training, validation, and test sets at a ratio of approximately 8:1:1. The training set contains 914 images, the validation set 113 images, and the test set 117 images, ensuring scientific rigor and reliability in model training and evaluation.

**Fig. 7:**
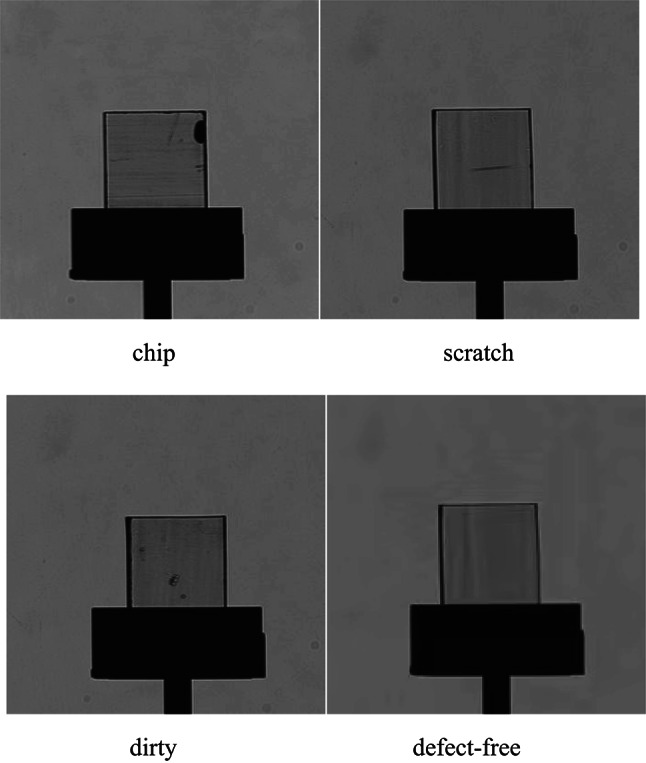
Glass defect data examples.

Regarding defect type distribution, the dataset covers three primary defect types commonly encountered in glass manufacturing: 303 chip defects, 310 dirty defects, and 358 scratch defects. To further evaluate the model’s discriminative ability between defective and non-defective glass under realistic industrial conditions, an additional 214 defect-free glass images were collected using the same shadowgraphy system. These images contain no scratches, chips, or contamination. The total dataset thus expanded to 1,358 images (1,144 defective + 214 defect-free). These 214 defect-free samples were split into training, validation, and test sets at a ratio of 8:1:1 (i.e., 171 for training, 21 for validation, and 22 for testing), and were incorporated into the corresponding splits alongside the original defective samples. This enriched dataset enables a more rigorous assessment of the model’s false positive rate and overall robustness.Examples are shown in Fig. 7.

This relatively balanced category distribution facilitates the model’s learning and recognition of different defect types, avoiding bias issues caused by data imbalance. The application of the optical observation system ensures high-resolution images and clear presentation of defect features, providing a high-quality data foundation for subsequent deep learning model training. This effectively supports the development and validation of glass defect detection algorithms.Simultaneously, processing training samples with both Mixup and Mosaic data augmentation strategies significantly enhances the model’s robustness and generalization capabilities.

To comprehensively evaluate the performance of the YOLO_FSA network in glass defect detection, this paper employs a multi-dimensional metric analysis. For detection accuracy, Precision measures the proportion of actual positive samples correctly predicted as positive^29^, while Recall assesses the model’s ability to identify all positive samples. These metrics effectively reflect the model’s accuracy and completeness in defect detection.Additionally, mean average precision (mAP50:95 and mAP50) serves as the core evaluation metric. mAP50 denotes the mean average precision at an IoU threshold of 0.5,while mAP50:95 denotes the mean average precision across IoU thresholds from 0.5 to 0.95 in 0.05 increments^30^. These metrics provide a more comprehensive assessment of the model’s detection performance under varying precision requirements.

Regarding model complexity assessment, statistical parameter counts measure storage requirements and memory consumption, while GFLOPS (gigaflops) evaluates computational complexity and inference efficiency. The combined use of these metrics not only evaluates the detection effectiveness of the YOLO_FSA network but also analyzes its practical applicability, providing crucial reference for engineering deployment.

### Ablation studies

#### Ablation study based on the defective dataset (excluding defective samples)

To validate the effectiveness of each improvement module, detailed ablation experiments were designed to analyze the contributions of the three core modules—C3k2_FWD, ADown, and GSCSA—to model performance, as shown in Table 2. Experiment A serves as the baseline model, employing the original YOLOv11 architecture. It achieves mAP50 of 0.729 and mAP50-95 of 0.466 on the glass defect detection task, with 2.583 million parameters and computational complexity of 6.3 GFLOPs. By progressively adding different improvement modules, the specific impact of each module on model performance can be clearly observed.

Regarding single-module improvements, Experiment B introduced the C3k2_FWD module, which slightly reduced mAP50 to 0.728 but significantly decreased parameters to 2.236 million and GFLOPs to 5.7,demonstrating its lightweighting advantage. Experiment C added the ADown module, boosting accuracy to 0.741 while further reducing parameters and computational load. The introduction of the GSCSA module in Experiment D achieved a recall rate of 0.758, though accuracy and mAP metrics declined. Multi-module combination experiments demonstrated more pronounced performance gains. Notably, Experiment H—integrating all three improvement modules—achieved optimal performance across all metrics: Accuracy reached 0.748, recall was 0.807, mAP50 improved to 0.855, and mAP50-95 reached 0.541. Meanwhile, the number of parameters was controlled at 1.77 million, and GFLOPs were only 4.7, fully validating the synergistic effects among modules and the effectiveness of the overall improvement scheme.

Experiment C added the ADown module, boosting the accuracy to 0.741 while further reducing the number of parameters and computational load, realizing the dual optimization of accuracy and lightweight. This indicates that the ADown module effectively retains the key features of glass defects and reduces feature loss by optimizing the downsampling strategy, thereby improving the detection accuracy of the model.

After introducing the GSCSA module in Experiment D, the recall rate reached 0.758, though the accuracy and mAP metrics declined. The reason is that the GSCSA module, as an attention mechanism, mainly focuses on enhancing the feature representation of defect areas and improving the recognition ability of hidden and weak-feature glass defects, thereby increasing the recall rate. However, it may also introduce some redundant features in non-defect areas, leading to a slight drop in accuracy and mAP metrics.

Multi-module combination experiments demonstrated more pronounced performance gains, which stems from the synergistic and complementary effects among the modules: the C3k2_FWD module is responsible for streamlining the model structure and reducing computational overhead, laying the foundation for model lightweight; the ADown module optimizes the efficiency of feature extraction, reduces feature loss, and improves detection accuracy; the GSCSA module enhances effective feature representation and improves the recognition ability of weak-target defects. The three modules work synergistically to effectively avoid the performance shortcomings of single-module application and achieve complementary advantages.

Notably, Experiment H—integrating all three improved modules—achieved optimal performance across all metrics: accuracy reached 0.748, recall was 0.807, mAP50 improved to 0.855, and mAP50-95 reached 0.541. Meanwhile, the number of parameters was controlled at 1.77 million, and GFLOPs were only 4.7, which fully validates the synergistic effects among the modules and the effectiveness of the overall improvement scheme, achieving the dual optimization goals of model accuracy and lightweight.

**Table 2:** Ablation study.

Algorithm	YOLOv11	C3k2_FWD	ADown	GSCSA	*P*	*R*	mAP50	mAP50-95	Parameters	GFLOPs
A	√	×	×	×	0.663	0.743	0.729	0.466	2,582,737	6.3
B	√	√	×	×	0.67	0.664	0.728	0.447	2,235,860	5.7
C	√	×	√	×	0.741	0.66	0.7	0.425	2,100,177	5.1
D	√	×	×	√	0.629	0.758	0.716	0.455	2,596,241	6.5
E	√	√	√	×	0.759	0.721	0.748	0.423	1,753,300	4.5
F	√	√	×	√	0.71	0.631	0.747	0.45	2,249,364	5.8
G	√	×	√	√	0.651	0.758	0.731	0.436	2,113,681	5.3
H	√	√	√	√	0.748	0.807	0.855	0.541	1,770,692	4.7

The above experimental results indicate that the three improved modules do not function independently; instead, they form a synergistic processing chain of “feature refinement—efficient extraction—attention enhancement” through functional complementarity. Specifically, C3k2_FWD performs local spatial mixing via partial convolution (only updating a fraction of channels), enhances defect-related channel responses using efficient channel attention (ECA), and aggregates multi-scale features with depthwise separable convolution. Its core role is to refine defect features and suppress background texture interference at the front end of the model, providing cleaner input for subsequent processing. However, when used alone (Experiment B), the partial convolution may insufficiently preserve the low‑frequency global contour information of defects such as chipping and contamination (since only a subset of channels is updated), while the ECA module could over‑suppress channels that are not strongly correlated with the current defect distribution. These effects together lead to a substantial drop in recall. The ADown module adopts a downsampling strategy using parallel average pooling and max pooling, preserving positional information while reducing spatial resolution, effectively aggregating neighborhood features and suppressing residual noise. Yet when used alone (Experiment C), low-resolution feature maps tend to lose small targets such as fine scratches and tiny stains, also harming recall. The GSCSA attention module, through multi-scale convolutions and dual spatial-channel weighting, can precisely localize defect regions and enhance weak feature responses. However, in the absence of the other two modules (Experiment D), the attention mechanism simultaneously amplifies background noise (e.g., optical artifacts, surface reflections), leading to a surge in false positives and a sharp drop in precision.

When all three modules are used together (Experiment H), the synergy is fully realized: C3k2_FWD pre-filters low-frequency noise and enhances defect edges; ADown then downsamples while more effectively retaining critical features and avoiding the loss of small targets; GSCSA finally performs spatial-channel reweighting on the enhanced feature maps to precisely localize defects. This process of “pre-filtering → downsampling preservation → attention focusing” exactly compensates for the weaknesses of each module when used alone: C3k2_FWD avoids excessive suppression of low-frequency information (compensated by ADown’s context retention), ADown avoids loss of small targets (compensated by GSCSA’s local response enhancement), and GSCSA avoids noise amplification (suppressed by C3k2_FWD’s pre-filtering). Consequently, the mAP50 of Experiment H (0.855) far exceeds those of Experiments E (0.748), F (0.747), and G (0.731), validating that synergistic compatibility among modules—rather than the mere stacking of advanced modules—is the key to achieving dual optimization of accuracy and lightweight design.

#### Ablation study on the extended dataset (including defect-free samples)

To evaluate the model’s ability to discriminate between defective and defect-free glass under realistic industrial conditions, we repeated the ablation experiments on the extended dataset that includes 214 additional defect‑free images (total 1,358 images). The training, validation, and test splits were kept identical to those described in Sect.  3.2. Table 3 presents the results of the eight ablation configurations on this extended dataset.

**Table 3:** Ablation study.

Algorithm	YOLOv11	C3k2_FWD	ADown	GSCSA	*P*	*R*	mAP50	mAP50-95	Parameters	GFLOPs
A	√	×	×	×	0.676	0.769	0.76	0.47	2,582,737	6.3
B	√	√	×	×	0.495	0.646	0.568	0.308	2,235,860	5.7
C	√	×	√	×	0.782	0.794	0.831	0.499	2,100,177	5.1
D	√	×	×	√	0.629	0.758	0.716	0.455	2,596,241	6.5
E	√	√	√	×	0.62	0.681	0.729	0.436	1,753,300	4.5
F	√	√	×	√	0.594	0.748	0.629	0.338	2,249,364	5.8
G	√	×	√	√	0.816	0.642	0.775	0.503	2,113,681	5.3
H	√	√	√	√	0.848	0.74	0.839	0.528	1,770,692	4.7

This paper introduces the use of defect-free samples to construct an extended dataset for comparative experiments, further enhancing the model’s detection and discrimination capabilities. The model is required not only to accurately identify genuine defects on glass surfaces but also to reliably distinguish defect-free glass regions. Compared to ablation experiments using only defect samples, the extended dataset more vividly demonstrates the complementary performance of each improved module and their respective limitations. The baseline model YOLOv11n achieves an mAP50 of 0.760, precision of 0.676, and recall of 0.769 on the extended dataset. The moderate precision indicates that the baseline model is prone to misjudging defect-free glass regions, resulting in numerous false positives—a common technical challenge in industrial glass defect detection tasks. The single-module ablation experiments revealed significant differences in the independent performance of various improved modules: When the C3k2_FWD module was introduced alone, all model metrics declined markedly, with mAP50 dropping to 0.568. This was attributed to the module’s lack of an appropriate downsampling architecture and attention mechanism, which led to excessive suppression of image features and misclassification of normal glass backgrounds as defects. Additionally, certain convolutional and ECA mechanisms erroneously suppressed low-frequency defect feature information, exacerbating false-negative rates. The ADown module alone achieved optimal single-module performance, with mAP50, recall rate, and precision reaching 0.831,0.794, and 0.782, respectively. By integrating parallel average pooling and maximum pooling downsampling strategies, this module effectively preserves spatial defect feature information while compressing feature resolution, demonstrating strong robustness against noise in non-defective samples. Although the GSCSA attention module increased recall to 0.758, precision decreased to 0.629, indicating that while the attention mechanism enhances defect localization accuracy, it amplifies background feature responses in non-defective regions, resulting in higher false-positive rates. In the dual-module combination experiments, significant differences in modular synergy were observed: The combined performance of C3k2_FWD with ADown failed to surpass that of the standalone ADown module, achieving an mAP50 of only 0.729—lower than the baseline model—indicating that ADown alone possesses exceptional feature extraction capabilities, while integrating C3k2_FWD introduces feature redundancy without enhancing detection accuracy. The combination of C3k2_FWD with GSCSA yielded the poorest overall performance, with mAP50 and precision values dropping to 0.629 and 0.594 respectively, confirming that C3k2_FWD cannot effectively synergize with the attention mechanism when operating without the downsampling module. While the ADown-GSCSA combination achieved a high precision of 0.816, its recall rate was only 0.642, reflecting effective suppression of false positives but severe false-negative rates—likely due to GSCSA’s spatial-channel attention mechanism directly processing ADown’s output features, resulting in excessive feature sensitivity. The YOLO_FSA model, integrating all three modules, delivered optimal performance with precision, recall, mAP50, and mAP50-95 values of 0.848,0.740,0.839, and 0.528 respectively, while requiring only 1.77 million parameters and 4.7 GFLOPs of computational resources, combining lightweight architecture with high precision. Compared to the standalone ADown module, the YOLO_FSA achieves a 6.6% point improvement in precision while maintaining only a slight decline in recall. When contrasted with the dual-module combination of ADown and GSCSA, this model demonstrates a 9.8% point increase in recall while preserving high precision. The outstanding performance stems from the synergistic optimization of three modules: C3k2_FWD employs partial convolutional and ECA mechanisms for image noise filtering while retaining critical defect features; ADown enables efficient downsampling while preserving spatial details; GSCSA enhances defect feature recognition through spatial and channel attention mechanisms, suppressing background noise interference. This three-stage feature optimization process addresses the inherent limitations of both single-module and dual-module approaches. Experimental results on extended datasets confirm that ADown serves as the core module ensuring the model’s resistance to interference in defect-free samples; standalone application of C3k2_FWD reduces detection performance, whereas multi-module collaboration achieves efficient noise suppression; independent use of GSCSA increases false positive rates, but its integration with the other two modules precisely enhances defect features while minimizing background noise interference. In conclusion, the proposed YOLO_FSA model exhibits low false positive rates and high detection accuracy, making it well-suited for industrial inspection scenarios with high proportions of defect-free glass samples and demonstrating strong practical engineering value.

#### k-fold cross-validation on the extended dataset

To more robustly evaluate the generalization capability of YOLO_FSA on an extended dataset containing defect-free samples and to mitigate the randomness introduced by a single random partition, we performed 5-fold cross-validation on 1,358 images (including 214 defect-free samples). The dataset was evenly divided into five parts, with four parts used for training and one for validation in each round. After five iterations, the mean and standard deviation of each metric were calculated. 

Table 4 presents a comparison of the overall performance of YOLOv11n and YOLO_FSA under 5-fold cross-validation.

**Table 4:** 5-fold cross-validation results on the extended dataset (including defect-free samples).

Algorithm	*P*(mean ± std)	*R*(mean ± std)	mAP50(mean ± std)	mAP50-95(mean ± std)
YOLOv11n	0.7782 ± 0.0600	0.6908 ± 0.0600	0.7764 ± 0.0350	0.4800 ± 0.0320
YOLO_FSA	0.8146 ± 0.0769	0.7454 ± 0.0671	0.8240 ± 0.0230	0.4940 ± 0.0238

As shown in Table 4, YOLO_FSA outperforms YOLOv11n across all core metrics, with standard deviations remaining within reasonable ranges, indicating that the improved model exhibits enhanced stability and generalization capabilities. Specifically, YOLO_FSA achieves an average precision (P) of 0.8146, a 3.6% point improvement over the baseline; an average recall (R) of 0.7454, a 5.5% point increase; an mAP50 of 0.8240, a 4.8% point improvement; and an mAP50-95 of 0.4940, a 1.4% point enhancement. Notably, YOLO_FSA reduces its parameter count by 31.4% (from 2.58 million to 1.77 million) and computational cost by 25.4% (from 6.3 GFLOPs to 4.7 GFLOPs), demonstrating the effective balance between lightweight architecture and detection accuracy achieved by the proposed combination of C3k2_FWD, ADown, and GSCSA modules.

In terms of standard deviation, the mAP50 standard deviation of YOLO_FSA (0.0230) is significantly lower than that of YOLOv11n (0.0350), indicating that the module-optimized model exhibits reduced sensitivity to different data splits and enhanced robustness. The standard deviations for precision and recall are slightly higher than those of the baseline, likely due to the inclusion of defect-free samples in the extended dataset. Although the background suppression performance of the GSCSA attention mechanism shows minor fluctuations across folds, it remains within an acceptable range overall. Comprehensive 5-fold cross-validation results demonstrate that YOLO_FSA improves detection accuracy while significantly reducing model complexity and inference costs, while maintaining strong cross-fold consistency—a testament to its potential for stable deployment in real-world industrial scenarios.

### Comparative experiments

To ensure the fairness of the comparative experiments and the validity of the ablation analysis, all models in this paper were trained and tested under the same hardware and software environment. All compared algorithms (including YOLOv5n through YOLOv13n, YOLO26n, RT-DETR-r18, and our YOLO_FSA) adopted the same training strategy, and the data augmentation was uniformly set to Mosaic and MixUp without introducing additional methods to avoid interference. Through this aligned setup, the performance differences reported in this paper can be reliably attributed to the architectural improvements of the model itself rather than to variations in the training environment or hyperparameters. On this basis, to comprehensively evaluate the performance advantages of the improved YOLO_FSA algorithm, we selected multiple mainstream object detection algorithms for comparative experiments, categorizing them into classic models, relatively new models, latest models, and high-parameter-count models. Among the classic lightweight models, YOLOv5n, as an early benchmark, has only 2.18 M parameters and 5.8 GFLOPs, but its performance—precision of 0.588 and mAP50 of 0.681—is relatively weak, making it suitable only for basic detection scenarios with extremely limited computational power. YOLOv8n, an iterative version of the classic models, achieves an mAP50 of 0.76 and mAP50-95 of 0.465 with similar model sizes (2.68 M parameters, 6.8 GFLOPs), striking a good balance between lightweight design and detection accuracy, and thus serving as a core benchmark for subsequent lightweight models. Among the relatively new lightweight models, YOLOv6n adopts a deeper network structure and decoupled head design, with parameters reaching 4.23 M and computational cost of 11.8 GFLOPs—significantly larger than other lightweight models—yet its mAP50 is only 0.658, indicating poor cost-effectiveness and weak adaptability to Schlieren glass defects. YOLOv12n and YOLOv13n, while continuing the lightweight approach with parameters controlled between 2.45 M and 2.51 M and computational cost on par with classic lightweight models, show a clear drop in accuracy. In particular, YOLOv13n achieves a recall of only 0.519 and an mAP50 of 0.635, a significant decline compared to YOLOv8n. The main reason is that both models prioritize inference speed and deployment simplification, sacrificing some detection accuracy by introducing lightweight modules. Among the latest lightweight models, YOLOv10n achieves a breakthrough in accuracy with its NMS-free end-to-end architecture, reaching an mAP50 of 0.792 and mAP50-95 of 0.509 with 2.27 M parameters and 6.5 GFLOPs, making it one of the best-performing lightweight models in terms of accuracy. YOLO26n, the newest model in the series, focuses on extreme lightweight design, with parameters of 2.38 M and computational cost of 5.2 GFLOPs—both the lowest among all models. Although its accuracy is moderate (mAP50 of 0.666, mAP50-95 of 0.384), it has outstanding CPU inference speed, making it suitable for low-power devices such as embedded systems. High-parameter-count models trade computational cost for higher detection accuracy. YOLOv9s has 7.17 M parameters and 26.7 GFLOPs; leveraging an efficient aggregation network design, it achieves a precision of 0.776 and an mAP50 of 0.788, making it suitable for high-accuracy scenarios with sufficient computational resources. RT-DETR-r18, a Transformer-based detector, has the highest computational demand among all models—19.88 M parameters and 56.9 GFLOPs—but also achieves top-tier performance with a precision of 0.881 and mAP50 of 0.817. Thanks to its global modeling capability, it attains the best detection accuracy but is only suitable for high-computing platforms such as GPU servers. Notably, the self-developed YOLO_FSA model, with the lowest parameters (1.77 M) and computational cost (4.7 GFLOPs) among all models, achieves the best overall accuracy in terms of recall (0.807), mAP50 (0.855), and mAP50-95 (0.541). Through feature fusion and attention mechanism optimization, it perfectly balances lightweight design, real-time performance, and detection accuracy, validating the effectiveness of the proposed optimization strategies. Overall, the iteration of lightweight models exhibits a pattern of “classic models for stability, newer models for efficiency, and the latest models for extreme lightweightness,” while high-parameter-count models continue to pursue high accuracy. The performance differences among these models provide a clear basis for algorithm selection under different computational scenarios (Table 5).

**Table 5:** Comparative experiment of mainstream algorithms.

Algorithm	*P*	*R*	mAP50	mAP50-95	Parameters	GFLOPs
YOLOv5n	0.588	0.738	0.681	0.424	2,182,249	5.8
YOLOv6n	0.692	0.715	0.658	0.397	4,234,041	11.8
YOLOv8n	0.676	0.724	0.76	0.465	2,684,953	6.8
YOLOv9s	0.776	0.734	0.788	0.499	7,168,249	26.7
YOLOv10n	0.713	0.686	0.792	0.509	2,265,753	6.5
YOLOv11n	0.663	0.743	0.729	0.466	2,582,737	6.3
YOLOv12n	0.729	0.614	0.695	0.396	2,508,929	5.8
YOLOv13n	0.727	0.519	0.635	0.368	2,448,480	6.2
RT-DETR-r18	0.881	0.719	0.817	0.509	19,875,612	56.9
YOLO26n	0.733	0.556	0.666	0.384	2,375,421	5.2
YOLO_FSA	0.748	0.807	0.855	0.541	1,770,692	4.7

### Detection performance analysis of different defect categories

To thoroughly evaluate the detection capability of the YOLO_FSA algorithm for various types of glass defects, we statistically calculated the Precision (P), Recall (R), mAP50, and mAP50-95 metrics for three defect categories—dirty, chip, and scratch—on the test set. A comparative analysis with the baseline YOLOv11n was also conducted, as presented in Table 6.

**Table 6:** Comparison of detection performance for different defect categories (YOLOv11n vs. YOLO_FSA).

Defect Type	Metric	YOLOv11n	YOLO_FSA	Absolute Gain	Relative Gain
Scratch	mAP50	0.747	0.973	+ 0.226	+ 30.3%
	mAP50-95	0.547	0.652	+ 0.105	+ 19.2%
	Recall	0.710	0.996	+ 0.286	+ 40.3%
	Precision	0.768	0.823	+ 0.055	+ 7.2%
Chip	mAP50	0.614	0.752	+ 0.138	+ 22.5%
	mAP50-95	0.417	0.529	+ 0.112	+ 26.9%
	Precision	0.473	0.876	+ 0.403	+ 85.2%
	Recall	0.714	0.714	0.0	0%
Dirty	mAP50	0.826	0.840	+ 0.014	+ 1.7%
	mAP50-95	0.433	0.441	+ 0.008	+ 1.8%
	Precision	0.748	0.846	+ 0.098	+ 13.1%
	Recall	0.806	0.710	-0.096	-11.9%

The most significant performance improvement was achieved for scratch defects: the mAP50 of YOLO_FSA increased from 0.747 to 0.973 (+ 30.3%), the mAP50-95 rose from 0.547 to 0.652 (+ 19.2%), the Recall improved from 0.710 to 0.996 (+ 40.3%), and the Precision grew from 0.768 to 0.823 (+ 7.2%). This result directly verifies the effectiveness of the partial convolution (for adaptive spatial mixing), efficient channel attention (for enhancing edge-related channels), and depthwise separable convolution (for multi-scale feature aggregation) embedded in the proposed C3k2_FWD module.

For chip defects, the performance gains are mainly reflected in Precision and mAP50-95: the Precision of YOLO_FSA increased substantially from 0.473 to 0.876 (+ 85.2%), the mAP50 increased from 0.614 to 0.752 (+ 22.5%), and the mAP50-95 improved from 0.417 to 0.529 (+ 26.9%). In shadow imaging systems, chip defects manifest as abrupt brightness changes in edge regions. The GSCSA attention mechanism enhances the perception of irregular shapes in edge regions through multi-scale feature fusion, and effectively reduces false detections with a sharp decline in false positives.

For dirty defects, the optimization is primarily reflected in Precision: the mAP50 of YOLO_FSA increased from 0.826 to 0.840 (+ 1.7%), and the Precision rose from 0.748 to 0.846 (+ 13.1%), while the Recall decreased from 0.806 to 0.710. Analysis indicates that dirty defects feature irregular morphologies and inconsistent contrast with the background. Some faint stains may be excessively suppressed during the noise amplification process of the GSCSA mechanism, or the confidence threshold setting leads to an increase in missing detections. Nevertheless, the mAP50 achieved a slight improvement and the Precision was significantly enhanced, demonstrating that the algorithm possesses a stronger ability to control false detections for dirty defects. Meanwhile, the mAP50-95 increased from 0.433 to 0.441, maintaining a stable performance.

In conclusion, the YOLO_FSA algorithm delivers performance improvements of varying degrees for all defect categories. In particular, it achieves an nearly perfect recall rate (99.6%) and a remarkable mAP50 (97.3%) for slender, low-contrast scratch defects. This fully validates the effectiveness of the improved modules proposed in this paper, which are tailored to the inherent characteristics of glass defects.

### Detection performance comparison

**Fig. 8:**
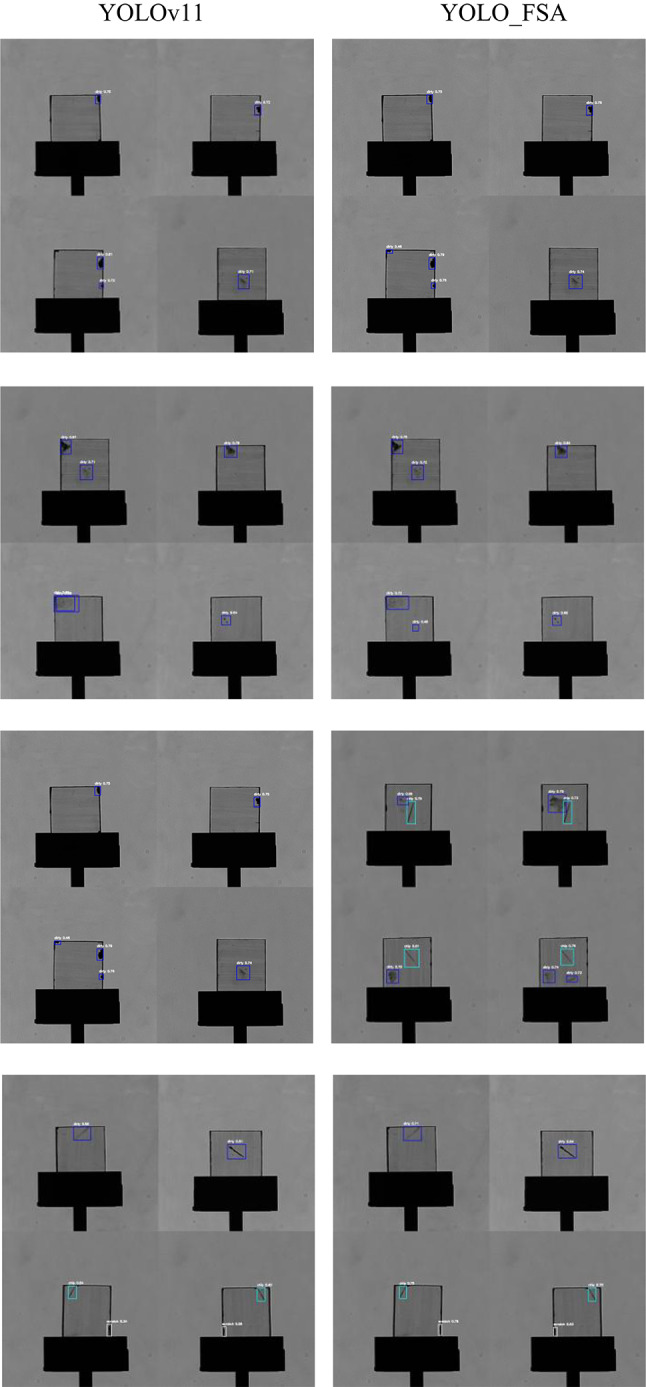
Glass defect detection performance comparison legend.

As shown in Fig. 8, the two sets of images demonstrate the performance comparison between the original YOLOv11n model and the improved YOLO_FSA model in glass defect detection. The left side displays the detection results of the original model, while the right side shows those of the YOLO_FSA model.

The right-hand images were detected using the improved YOLO_FSA algorithm. Various defect types, such as scratches and stains, are accurately identified with corresponding confidence scores. In contrast, the YOLOv11n model on the left exhibits limitations in detecting elongated scratches and stains with blurred edges. Its defect classification accuracy and confidence assessment fall short of the YOLO_FSA algorithm.

This demonstrates that the YOLO_FSA algorithm, incorporating the proposed improvements C3k2_FWD, GSCSA, and ADown, can more effectively process low-contrast, blurred-edge glass defect images acquired under holographic optical systems, significantly enhancing detection performance. This result validates the effectiveness and advantages of the proposed improvements in addressing object detection challenges within specific industrial application scenarios.

### Discussion on industrial application impact

The proposed YOLO_FSA algorithm achieves a precision of 74.8% and a recall of 80.7% on the glass defect detection task. The precision corresponds to a false positive rate of approximately 25.2% (qualified glass misclassified as defective), while the recall corresponds to a false negative rate of approximately 19.3% (defective glass misclassified as qualified). In industrial glass quality control, the economic consequences of false positives and false negatives differ significantly.

For glass manufacturing, false negatives generally incur higher costs—defective products reaching customers may lead to quality complaints, returns, or even safety hazards. In contrast, false positives have a relatively lower cost, as misclassified glass panels typically undergo secondary manual inspection rather than direct scrapping. Therefore, prioritizing recall (i.e., minimizing missed defects) is a reasonable engineering choice. The recall of 80.7% means that approximately four-fifths of actual defects are automatically detected, while the remaining one-fifth of missed defects need to be further intercepted by subsequent processes (e.g., manual reinspection or supplementary algorithms).

It should be noted that a false negative rate of 19.3% may still be considered high for some high‑standard production lines. To further reduce the impact of missed defects while controlling the re‑inspection burden caused by false positives, two practical strategies can be adopted: (1) Confidence threshold adjustment—lowering the detection confidence threshold during deployment can further improve recall (at the cost of increased false positives), suitable for scenarios requiring near‑zero missed defects; (2) Two‑stage detection pipeline—using YOLO_FSA as a high‑recall screener, followed by a lightweight verification classifier to re‑examine samples initially classified as “qualified”, thereby capturing defects missed in the first stage. Future work will explore these deployment strategies and conduct a more refined trade‑off analysis between false negatives and false positives based on real‑world production line data, thereby enhancing the industrial applicability of the algorithm.

### Discussion and limitations

Although the proposed method achieves promising preliminary results on the Schlieren-based glass defect detection task, several limitations remain.

First, the dataset size is limited. Due to the stringent requirements of the Schlieren system on optical environment and equipment precision, as well as the unavailability of the experimental equipment during the repair period (deadline April 7), this study collected only 1,144 valid images. For a deep learning model with a large number of parameters such as YOLOv11, this dataset size is relatively small, which may to some extent affect the model’s generalization ability and statistical significance. Although we employed strong data augmentation strategies (Mosaic and MixUp) to alleviate the small-sample issue, the risk of overfitting cannot be completely eliminated.

Second, there is a lack of publicly available benchmark datasets. To the best of our knowledge, no publicly available Schlieren-based glass defect dataset exists; therefore, the proposed method cannot be directly compared with existing approaches on identical data. The generalizability of our conclusions remains to be validated on larger and more diverse datasets.

Third, there is a trade-off between model complexity and real-time performance. The integration of multiple enhancement components to improve detection accuracy inevitably increases computational overhead. For strict real-time requirements in practical industrial scenarios, the model may require further lightweight compression or distillation.

Fourth, the coverage of defect types is incomplete. The current dataset mainly includes common glass defects such as scratches, bubbles, and stains, but does not cover all possible defect types (e.g., cracks, stones). The model’s ability to recognize rare defects remains unclear.

In addition, this study currently focuses on the fine-grained classification of glass surface defects, aiming to achieve accurate categorization of pre-screened suspected defective samples in industrial inspection pipelines. In practical industrial scenarios, a complete defect detection system usually consists of a front-end binary classification module (distinguishing between normal and defective samples) and a back-end fine-grained classification module (identifying defect types). The proposed method is positioned as the core back-end module, which performs refined classification of suspected defects and provides support for product quality grading. Limited by the collection scope of the current dataset, this study does not include a large number of defect-free glass samples, so the training and evaluation of the binary classification task between normal samples and defective samples are not conducted. To address this limitation, we clarify that the proposed method can be seamlessly integrated with front-end modules such as traditional threshold segmentation and deep learning binary classifiers to form an end-to-end industrial defect detection system.

To address the above limitations comprehensively, we plan the following future work: collect more images (target > 5,000) once the Schlieren equipment becomes available again and consider releasing the dataset publicly; explore semi-supervised, self-supervised, or few-shot learning paradigms to reduce dependence on large-scale annotated data; perform lightweight model optimization for edge deployment; collect more types of glass defect images to improve generalization coverage; and supplement a large-scale defect-free sample dataset, improve the binary classification ability between normal samples and defective samples, and realize full-process detection from raw industrial samples to defect type identification, so as to further enhance the practical applicability of the method.

## Conclusion

This paper addresses the challenge of detecting low-contrast, blurred-edge defects on glass surfaces under a shadowgraphy imaging system by proposing the YOLO_FSA algorithm. Built upon the YOLOv11n baseline, the algorithm integrates three core modules: C3k2_FWD (combining partial convolution, efficient channel attention (ECA), depthwise separable convolution, and hierarchical residual fusion), GSCSA (a glass-oriented spatial-channel self-attention mechanism), and ADown (a lightweight downsampling module). Experimental results show that YOLO_FSA achieves precision, recall, and mAP50 of 74.8%, 80.7%, and 85.5%, respectively, which are improvements of 8.5, 6.4, and 12.6% points over YOLOv11n. Meanwhile, the number of parameters is reduced to 1.77 M (a decrease of 31.4%) and computational complexity to 4.7 GFLOPs (a decrease of 25.4%), achieving a balance between detection performance and computational efficiency.

From the perspective of scientific contribution, this work has two main theoretical values:

First, it validates the effectiveness of joint local spatial mixing and channel-wise attention for low-contrast tiny defects. Glass defects (especially scratches) appear as weak edge information in the shadowgraphy imaging system, which conventional spatial convolutions cannot fully capture. The C3k2_FWD module uses partial convolution (Eq. 1) to split features into a convolution-processed branch and an identity branch, preserving spatial details while reducing redundancy. The ECA mechanism (Eq. 2) then adaptively calibrates channel responses, enhancing the high-frequency defect boundaries and suppressing background textures. Depthwise separable convolution (Eq. 3) further aggregates multi-scale features with minimal computational cost. This explicit strategy of “local mixing, then channel recalibration, then multi-scale aggregation” provides a new solution for the common “weak signal, strong noise” object detection problem in industrial vision, differing from simply stacking convolutional layers.

Second, it reveals the synergistic adaptation principle between attention mechanisms and lightweight downsampling. The ablation study (Table 2) shows that introducing the GSCSA module alone (Experiment D) reduces precision from 0.663 to 0.629 and mAP50 from 0.729 to 0.716; introducing ADown alone (Experiment C) also reduces mAP50 to 0.700. However, when all three modules are used together (Experiment H), all metrics reach their best values (mAP50 of 0.855). The underlying mechanism of this non-additive phenomenon is as follows: the multi-scale convolutions and dual attention of GSCSA enhance defect signals but also amplify background noise; ADown, through parallel average pooling and max pooling downsampling, preserves spatial location information while reducing resolution, thereby aggregating features and suppressing noise; C3k2_FWD pre-filters interference using partial convolution and ECA as a “local-channel” denoising front-end. The three together form a synergistic chain of “local-channel denoising, then noise-suppressing downsampling, then attention focusing”. The performance drops in Experiments D and C precisely indicate that introducing complex modules alone disrupts the original network’s gradient balance or loses detailed features; only through complementary functionality among modules can their respective potentials be unleashed. This finding suggests that in lightweight model design, synergistic compatibility among modules is more critical than the advanced nature of any single module.

From the perspective of practical engineering deployment and cost-benefit analysis, YOLO_FSA demonstrates clear industrial potential:

Computational efficiency and hardware compatibility: With only 1.77 M parameters and 4.7 GFLOPs, the model can be deployed on resource-constrained industrial edge devices (e.g., embedded GPUs or edge AI boxes) while maintaining near-real-time inference speeds. This low computational footprint reduces hardware procurement and energy costs, making the algorithm suitable for large-scale, multi-camera production lines.

Economic trade-offs between false positives and false negatives: In glass manufacturing quality control, false negatives (defective panels misclassified as qualified) typically incur higher costs due to potential customer complaints, product recalls, and safety hazards. Conversely, false positives (qualified panels flagged as defective) can be managed through secondary manual inspection or a re-checking mechanism. The proposed YOLO_FSA achieves a recall of 80.7%, significantly reducing the risk of missed defects compared to the baseline. The precision of 74.8% implies a moderate false-positive rate, which can be economically handled by downstream quality assurance processes without excessive labor burden. The modular design also allows operators to adjust the detection confidence threshold according to specific production line requirements, enabling flexible optimization of the false-positive/false-negative trade-off.

Workflow integration and ROI rationale: The algorithm naturally fits a “primary AI screening plus targeted manual re-inspection” workflow. By prioritizing recall through a slightly lowered confidence threshold, the majority of actual defects are automatically flagged, while false positives are efficiently re-verified by human inspectors. This hybrid scheme reduces the overall inspection cost compared to pure manual inspection and improves consistency compared to purely automated systems with high false-positive rates. The reduction in missed defects lowers the risk of downstream quality incidents, providing a compelling return-on-investment justification for glass manufacturers.

Future work will focus on two directions: (1) exploring more efficient variants of partial convolution or lightweight attention mechanisms (e.g., re-parameterized ECA) to further improve the detection rate of weak scratches while maintaining the already low parameter count; (2) introducing few-shot learning or domain adaptation techniques to quickly achieve cross-production-line migration for different glass substrates (e.g., ultra-clear glass, patterned glass), avoiding large-scale repeated data collection.

## Supplementary Information

Below is the link to the electronic supplementary material.

Supplementary Material 1

Supplementary Material 2

## Data Availability

The datasets used and analysed during the current study are available from the corresponding author on reasonable request.
