# Supplementary material for: Research on glass surface defect detection method based on shadowgraphy imaging and improved YOLOv11n
Source: Sci Rep. 2026 Jun 4;16:25440. doi: 10.1038/s41598-026-56410-y (PMC13473479; doi:10.1038/s41598-026-56410-y)
Supplement: Supplementary file 1 [file 41598_2026_56410_MOESM1_ESM.zip › docs/overrides/main.html]

{% extends "base.html" %} {% block announce %}

YOLO Vision 2024 is here!

September 27, 2024

Free hybrid event

Register now

{% endblock %}
